# Prevalence and Correlates of Diabetic Peripheral Neuropathy in a Saudi Arabic Population: A Cross-Sectional Study

**DOI:** 10.1371/journal.pone.0106935

**Published:** 2014-09-03

**Authors:** Dong D. Wang, Balkees A. Bakhotmah, Frank B. Hu, Hasan Ali Alzahrani

**Affiliations:** 1 The Departments of Nutrition, Harvard School of Public Health, Boston, Massachusetts, United States of America; 2 The Mohammad Hussein Al Amoudi Chair for Diabetic Foot Research, and the Department of Nutrition and Food Sciences, Art and Design College, King Abdulaziz University, Jeddah, Saudi Arabia; 3 The Departments of Nutrition, and Epidemiology, Harvard School of Public Health, Boston, Massachusetts, United States of America; 4 The Channing Division for Network Medicine, Department of Medicine, Brigham and Women's Hospital and Harvard Medical School, Boston, Massachusetts, United States of America; German Diabetes Center, Leibniz Center for Diabetes Research at Heinrich Heine University Duesseldorf, Germany

## Abstract

The purpose of this cross-sectional study was to investigate the prevalence and correlates of diabetic peripheral neuropathy (DPN) in a Saudi population. The study population consisted of 552 diabetic participants with an average age of 53.4 years. Among this population, 62.7% were male and 94.9% had type 2 diabetes. The average body mass index was 31.1 kg/m^2^. DPN was diagnosed based on a combination of reduced vibration perception measured by neurothesiometer and/or reduced light touch perception evaluated by the 10-g Semmes-Weinstein monofilament, as well as neurological symptoms. Information on socio-demographic variables, smoking status, duration of diabetes, and medications was obtained through interviews by physicians. Body weight, height, waist circumference, blood pressure and clinical markers were assessed following standard procedures. The prevalence of DPN in this population was 19.9% (95% CI, 16.7%-23.5%). In the multivariable analyses, longer duration of diabetes [odds ratio (OR) for every 5-year increase, 2.49, 95% CI, 1.75-3.53], abdominal obesity (OR, 2.53, 95% CI, 1.41-4.55), and higher levels of fasting blood glucose (OR for every 1 mmol/L increase, 1.05, 95% CI, 0.99-1.12), creatinine (OR for every 10 µmol/L increase, 1.07, 95% CI, 0.99-1.14) and white blood cell count (OR for every 10^6^/L increase, 1.08, 95% CI, 1.01-1.16) were associated with higher odds of DPN, while oral hypoglycemic medication use was associated with a lower odds of DPN (OR, 0.47, 95% CI, 0.26-0.85). In this large Saudi population, several correlates for DPN, in addition to glycemic control and diabetes duration, were identified, including abdominal obesity, creatinine and white blood cell count.

## Introduction

As a common complication of diabetes, diabetic peripheral neuropathy (DPN) is associated with a wide range of clinical manifestations, and predicts foot ulcer, lower-extremity amputation and mortality in diabetic patients [1], accounting for a massive amount of economic burden to healthcare system [2]. In addition, DPN is one of the most insidious complications of diabetes. Up to 50% of patients with DPN are asymptomatic but may already develop insensate foot injury [1]. Therefore, early recognition of high-risk population is enormously important so that rigorous modification of risk factors, accompanying foot care, could be implemented before or at early stage of neuropathic process, which will prevent the occurrence and improve the prognosis of DPN. However, according to the latest statement regarding DPN from the American Diabetes Association (ADA), other than strict blood glucose control, no prevention and treatment measures for DPN were recommended due to the lack of reliable scientific evidence [1]. Furthermore, the Diabetes Control and Complications Trial found that, even under rigorous blood glucose control, patients still presented substantially high cumulative incidence of DPN, suggesting that risk factors apart from blood glucose level could play an important role in the development of DPN [3]. Existing evidence suggests duration and level of hyperglycemia [3], [4], dyslipidemia, body mass index (BMI), smoking, hypertension [5] and height [6] as risk factors for DPN, but overall evidence is still limited.

Due to the dramatic changes on lifestyle and diet in the past decades, by 2011, Saudi Arabia (SA) has ranked as the country with the 7th highest adult diabetes prevalence (24.0%) around the world [7]. Furthermore, the prevalence of DPN in Middle-Eastern diabetic populations is relatively high. For example, in Bahrain, a neighboring country of SA, a DPN prevalence of 36.6% has been reported in a large diabetic population [8], comparable to that in U.S. diabetic populations (12% – 50%) [2]. Although the high prevalence of both diabetes and its complications threatens to overburden the health care system and create insurmountable public health challenges in SA, few studies have addressed potential risk factors of DPN in Saudi populations, which could be different from those already reported in other populations, because diabetic population in the Middle East usually have poorer glycemic control compared to those in developed countries [9]. In addition, different genetic and environmental backgrounds in diabetic populations could also contribute to different pattern of correlates of DPN [10]. Therefore, identifying correlates for DPN, as potential risk predictors, in order to facilitate DPN management is of great public health importance in SA [11].

In this present study, we investigated the prevalence and correlates for DPN among a diabetic population in Western SA.

## Methods

From June 2009 to May 2010, we published the invitation to participate our study in a widely read newspaper in Jeddah Governance, Saudi Arabia for recruitment of potential participants. Respondents to our invitation contacted our study staff through telephone to confirm their first clinical visit. During the first visit, they were screened by a questionnaire and clinical examinations based on inclusion and exclusion criteria. The inclusion criteria were age of 30 years or older and the history of diabetes of 2 years or longer. The exclusion criterion was presence of foot ulcer. The final study population was comprised of 552 participants. Compared to a nationally representative sample of diabetic patients for estimating the prevalence of DPN, our study population had similar demographic characteristics [12]. The study was approved by the King Abdulaziz University Hospital (KAUH) Ethical Committee. Study methods, benefits and adverse reactions, and objectives of this study were explained to all participants. Written consent was obtained from every participant. The diagnosis and type of diabetes mellitus was first self-reported by participants and then confirmed by physicians using medical records. A questionnaire was administered in person by a vascular specialist and a trained nurse to collect information on nationality, age, smoking status, personal income level, educational attainment, current medication use and duration of diabetes. Nationality was classified as Saudi and non-Saudi. Smoking status was classified as ever smoker and never smoker. Personal income level was categorized as <3000, 3000–10000 and >10000 Saudi Riyal (SR)/month (1 USD  = 3.75 SR). We categorized education attainment as illiterate, high school, university, and post-graduate. Duration of diabetes was categorized into 2–5, 5–10, 10–20 and >20 years.

The participant's body weight was measured to the nearest of 0.1 kg by an electronic weighing scale (Seca, Birmingham, United Kingdom). Height was measured without shoes to the nearest of 0.5 cm using a stadiometer (Seca, Birmingham, United Kingdom). Body mass index (BMI) was calculated as weight (kg) divided by height (m) squared (kg/m^2^). Waist circumference was measured at the smallest circumference between the rib margin and iliac crest. The cutoff points of BMI recommended by the World Health Organization (WHO) were used to define obesity (≥30 kg/m^2^) [13]. Abdominal obesity was defined as waist circumference ≥102 cm for men and ≥88 cm for women. All participants' blood pressures were measured by an electronic vital signs monitor (SuresignsVs3, Philips medical system, Andover, MA, USA). Two consecutive readings of blood pressure were taken in the right arm of participants in a seated position after 5 minutes of rest. The mean of the 2 measures was used for analysis. In this study, hypertension was defined as systolic blood pressure (SBP) ≥140 mmHg and/or diastolic blood pressure (DBP) ≥90 mmHg according to the Seventh Joint National Committee on Prevention, Detection, Evaluation, and Treatment of High Blood Pressure guidelines [14], and/or using the blood-pressure-lowering drugs. In our study, the diagnosis of DPN was based on a combination of both decreased sensation (reduced vibration perception and/or reduced light touch perception in either foot) and neuropathic sensory symptoms (loss of pinprick sensations, tingling, and deformed foot). Vibration perception was assessed over bony prominences on dorsal aspect of the 1st and 5th metatarsal heads using the Horwell Neurothesiometer (Scientific Laboratory Supplies, Nottingham, United Kingdom). Reduced vibration perception was defined by a vibration perception threshold ≥25 Volts in either foot [15]. Light touch perception was evaluated using a 10-g Semmes-Weinstein monofilament (Huntleigh Diagnostics, Cardiff, United Kingdom) at four sites of the foot (the plantar and dorsal aspect of 1st and 5th metatarsal heads) The participant should close his/her eyes when being tested and then recognize the perception of the pressure at correct site. Areas of callus were avoided when testing. Loss of perception at any of the four sites was defined as reduced touch perception. [16], [17]. Pinprick sensation test was performed by using a disposable pin with just enough pressure to deform the skin on the dorsal surface of the hallux. Loss of pinprick sensation was defined as failure to perceive pinprick over either hallux. [17] All the assessments were conducted by an assessor first and then confirmed by a second assessor independently.

Participants were instructed to fast and abstain from vigorous exercise for 12 hours prior to medical examination and blood draw. Fasting venous blood samples (10 ml) were taken from antecubital vein by the laboratory staff and sent to the KAUH's accredited central laboratory for daily assay. Fasting blood glucose, HbA1c [18], lipids profile [high-density lipoprotein (HDL), low-density lipoprotein (LDL), triglyceride (TG)] [19], homocysteine [20], creatinine [21], high sensitivity C-reactive protein (CRP) [22], serum urea nitrogen, hemoglobin, and complete blood count were measured using standard methods.

### Statistical analyses

The distributions of continuous variables were presented as median and interquartile range. A comparison of continuous variables between DPN and non-DPN participants was done by quantile regression with adjustment for age, sex and nationality when applicable. All the categorical variables were presented as number and percentage. For the clinical marker data, there were some missing values due to laboratory technical failure or participants' refusal to provide blood samples. Logistic regression model adjusted for age, sex and nationality was employed to compare the distribution of categorical variables between DPN and non-DPN participants. Less than 10% of clinical marker measurements were missing except for LDL (10.9%), homocysteine (26.8%) and CRP (11.2%). We therefore used the median value of each clinical marker to impute the missing values. Logistic regression model was used to examine the association between correlates and DPN. The first logistic regression model (model 1) estimated odds ratio (OR) and its 95% confidence interval (CI) for each correlate with adjustment for sex, age (continuous) and nationality (Saudi, non-Saudi). In the model 1, all the continuous variables were categorized into quartiles. To further explore significant independent correlates for DPN, we used a backward selection algorithm beginning with a model including all the potential correlates listed in Table 1. Sex and age were forced to be included in the model. A variable was ultimately retained in the final model if its P value ≤0.1 to accommodate the selection of important correlates. Model 2 examined the association between each selected independent correlates and DPN with adjustment for other independent correlates. In the sensitivity analysis, we repeated the same backward selection algorithm but excluded participants with type 1 diabetes from our analytical population. All statistical analyses were conducted using SAS 9.2 software (SAS Institute, Cary, NC). All *P*-values were 2-tailed (α  = 0.05, except the α for backward elimination).

**Table 1:** Characteristics of participants according to diabetic peripheral neuropathy status.

.	Non-DPN case	DPN case	*P* †
	(N = 441)	(N = 110)	
Sex, male ‡	278 (62.9)	68 (61.8)	0.577
Nationality, Saudi	273 (61.8)	60 (54.5)	0.044
Education			0.202
Illiterate	160 (36.2)	53 (48.2)	
High school	121 (27.4)	24 (21.8)	
University	133 (30.1)	27 (24.5)	
Post graduate	28 (6.3)	6 (5.5)	
Income level (SR/month)			0.232
<3000	176 (39.8)	54 (49.1)	
3000–10000	162 (36.7)	36 (32.7)	
>10000	104 (23.5)	20 (18.2)	
Obesity	215 (48.6)	62 (56.4)	0.055
Abdominal obesity	266 (60.2)	87 (79.1)	<.001
Smoking status, ever smoker	90 (20.4)	21 (19.1)	0.661
Type of diabetes, type 2	422 (95.7)	102 (92.7)	0.011
Duration of diabetes (years)			<.001
2–5	132 (29.9)	8 (7.3)	
5–10	120 (27.1)	11 (10.0)	
10–20	135 (30.5)	37 (33.6)	
>20	55 (12.4)	54 (49.1)	
Hypertension	270 (61.1)	86 (78.2)	0.017
Insulin	134 (30.3)	68 (61.8)	<.001
Oral hypoglycemic	376 (85.1)	76 (69.1)	<.001
Aspirin	250 (56.6)	75 (68.2)	0.675
Plavix	27 (6.1)	14 (12.7)	0.360
Statins	159 (36.0)	54 (49.1)	0.044
Age (years) §	52.0 (46.0–58.0)	59.5 (50.0–66.0)	<.001
Body mass index (kg/cm^2^)	29.7 (26.8–34.1)	30.8 (27.4–36.6)	0.246
Height (cm)	164.3 (157.0–172.0)	164.0 (158.0–172.0)	0.083
Waist circumference (cm)	100 (93–108)	105 (96–115)	0.001
Fasting blood glucose (mmol/L)	9.4 (7.7–12)	9.7 (7.9–13.9)	0.002
HbA1c (%)	9.0 (8.0–10.6)	9.0 (8.0–11.0)	0.147
HDL (mmol/L)	1.1 (1.0–1.3)	1.1 (1.0–1.3)	0.293
LDL (mmol/L)	3.0 (2.5–3.7)	3.0 (2.5–3.7)	0.755
Triglyceride (mmol/L)	1.5 (1.0–2.0)	1.5 (1.1–2.1)	0.184
Homocysteine (ìmol/L)	7.7 (6.4–8.9)	7.7 (7.7–9.7)	0.164
Creatinine (ìmol/L)	78.0 (64.0–88.0)	82.0 (75.0–103.0)	0.041
CRP (mg/L)	3.3 (3.2–5.8)	3.4 (3.3–9.5)	0.058
Serum urea nitrogen (mmol/L)	5.0 (4.0–5.9)	5.5 (4.7–7.7)	0.097
Hemoglobin (g/dL)	13.8 (12.8–14.9)	13.6 (12–14.5)	0.093
White blood cell (10^6^/L)	7.1 (5.9–8.5)	7.5 (6.4–9.5)	0.041
Platelet (10^9^/L) )	252.5 (219.0–294.0)	252.5 (205.0–285.0)	0.084

Abbreviations: DPN, diabetic peripheral neuropathy; SR, Saudi riyal (currency unit of Saudi Arabia); HbA1c, glycated hemoglobin; HDL, high-density lipoprotein cholesterol; LDL, low-density lipoprotein cholesterol; CRP, C-reactive protein.

† *P*-values were calculated from logistic regression models for categorical variables or quantile regression for continuous variables adjusted for age, sex and nationality when applicable.

‡ n (%) for all such values

§ median (25% percentile – 75% percentile) for all such values

## Results

Table 1 shows characteristics of the study population according to whether DPN was diagnosed or not. Among the 552 participants, 110 were diagnosed as DPN, representing a prevalence of 19.9% [95% confidence interval (CI), 16.7% – 23.5%]. The mean age of this population was 53.4±10.5 years. Participants with DPN were older, had longer duration of diabetes, and higher prevalence of abdominal obesity and hypertension, compared with their counterparts without DPN. Insulin was more commonly used in participants with DPN, while oral hypoglycemic and statin were more commonly used in participants without DPN (Table 1). Participants with DPN had significantly higher levels of waist circumference, fasting blood glucose, creatinine and white blood cell (WBC) count when compared to participants without DPN.

Table 2 shows the associations between the correlates and DPN after adjusting for age, sex and nationality. Abdominal obesity, type 1 diabetes, hypertension and insulin use were significantly associated with higher odds of DPN. A significant and positive association between duration of diabetes and odds of DPN was observed (P _trend_ <0.001). Participants with duration of diabetes >20 years presented a 12.19-fold odds of DPN (OR, 12.19, 95% CI, 5.24–28.36) when compared with those with duration of diabetes of 2–5 years. In oral hypoglycemic users, a significantly lower odds of DPN was observed. For clinical markers, higher levels of homocysteine, creatinine, CRP, serum urea nitrogen and WBC were significantly associated with higher odds of DPN, whereas a significantly lower odds of DPN was found among participants with higher hemoglobin level.

**Table 2:** Association between risk factors and prevalence of diabetic peripheral neuropathy among individuals with diabetes. †

	Cases (%)	Odds ratio (95% CI)	*P* ‡
Education			0.770
Illiterate	53 (24.9)	Reference	
High school	24 (16.6)	0.97 (0.54–1.76)	
University	27 (16.9)	0.90 (0.51–1.59)	
Post graduate	6 (17.6)	0.97 (0.36–2.63)	
Income level (SR/month)			0.702
<3000	54 (23.5)	Reference	
3000–10000	36 (18.2)	0.97 (0.56–1.68)	
>10000	20 (16.1)	0.86 (0.43–1.73)	
Obesity	62 (22.4)	1.57 (1.00–2.48)	0.052
Abdominal obesity	87 (24.6)	2.65 (1.55–4.56)	<.001
Smoking, ever-smoker vs. never-smoker	21 (18.9)	1.17 (0.66–2.08)	0.602
Type of diabetes, type 1 vs. type 2	102 (19.5)	4.08 (1.58–10.57)	0.004
Duration of diabetes (years)			<.001
2–5	8 (5.7)	Reference	
5–10	11 (8.4)	1.48 (0.57–3.83)	
10–20	37 (21.5)	3.80 (1.68–8.60)	
>20	54 (49.5)	12.19 (5.24–28.36)	
Hypertensive vs. normotensive	86 (24.2)	1.79 (1.07–2.99)	0.025
Insulin, user vs. non-user	68 (33.7)	3.93 (2.48–6.22)	<.001
Oral hypoglycemic, user vs. non-user	76 (16.8)	0.31 (0.19–0.53)	<.001
Aspirin, user vs. non-user	75 (23.1)	1.25 (0.78–1.99)	0.350
Plavix, user vs. non-user	14 (34.1)	1.78 (0.87–3.64)	0.114
Statins, user vs. non-user	54 (25.4)	1.51 (0.97–2.34)	0.067
Height (cm)			0.113
<157.5	27 (19.6)	Reference	
157.5–164.0	32 (22.5)	1.39 (0.72–2.69)	
164.0–172.0	22 (16.7)	1.28 (0.55–2.95)	
≥172.0	29 (20.7)	2.10 (0.88–5.03)	
Fasting blood glucose (mmol/L)			0.053
<7.7	26 (19.1)	Reference	
7.7–9.4	27 (16.8)	0.91 (0.49–1.68)	
9.4–12.5	24 (20.5)	1.17 (0.61–2.23)	
≥12.5	33 (23.9)	1.64 (0.90–3.02)	
HbA1c (%)			0.338
<8.0	27 (21.1)	Reference	
8.0–9.0	16 (15.5)	0.65 (0.32–1.32)	
9.0–10.6	37 (20.1)	0.99 (0.55–1.78)	
≥10.6	30 (21.9)	1.20 (0.65–2.23)	
HDL (mmol/L)			0.881
<1.0	30 (21.7)	Reference	
1.0–1.1	17 (15.2)	0.63 (0.32–1.24)	
1.1–1.3	35 (21.3)	0.98 (0.55–1.74)	
≥1.3	28 (20.3)	0.94 (0.51–1.76)	
LDL (mmol/L)			0.717
<2.5	28 (20.4)	Reference	
2.5–3.0	27 (25.2)	1.56 (0.83–2.94)	
3.0–3.7	28 (16.5)	1.09 (0.59–2.02)	
≥3.7	27 (19.6)	1.21 (0.65–2.24)	
Triglyceride (mmol/L)			0.138
<1.0	25 (18)	Reference	
1.0–1.5	28 (17.9)	1.02 (0.55–1.89)	
1.5–2.0	25 (21.2)	1.18 (0.62–2.25)	
≥2.0	32 (23)	1.51 (0.82–2.77)	
Homocysteine (ìmol/L)			0.087
<6.6	14 (9.9)	Reference	
6.6–7.7	9 (14.8)	1.44 (0.58–3.62)	
7.7–9.0	53 (24.9)	2.38 (1.24–4.58)	
≥9.0	34 (24.8)	2.15 (1.06–4.34)	
Creatinine (ìmol/L)			<.001
<66.0	15 (10.5)	Reference	
66.0–78.0	29 (18.4)	2.30 (1.14–4.65)	
78.0–90.0	23 (20.9)	2.52 (1.17–5.41)	
≥90.0	43 (30.5)	3.69 (1.76–7.75)	
CRP (mg/L)			<.001
<3.2	18 (13.0)	Reference	
3.2–3.3	23 (20.7)	1.74 (0.86–3.55)	
3.3–6.8	29 (17.6)	1.73 (0.88–3.40)	
≥6.8	40 (29.0)	3.55 (1.81–6.98)	
Serum urea nitrogen (mmol/L)			0.002
<4.1	15 (10.9)	Reference	
4.1–5.0	21 (18.6)	1.69 (0.81–3.54)	
5.0–6.1	29 (17.6)	1.75 (0.87–3.50)	
≥6.1	45 (33.1)	2.92 (1.47–5.79)	
Hemoglobin (g/dL)			0.011
<12.7	39 (28.5)	Reference	
12.7–13.8	28 (17.4)	0.51 (0.27–0.96)	
13.8–14.8	28 (23.7)	0.73 (0.37–1.43)	
≥14.8	15 (11.0)	0.32 (0.15–0.72)	
White blood cell (10^6^/L)			0.003
<5.9	19 (13.6)	Reference	
5.9–7.1	22 (19.6)	1.51 (0.76–3.02)	
7.1–8.7	28 (17.0)	1.35 (0.70–2.58)	
≥8.7	41 (30.4)	2.58 (1.37–4.85)	
Platelet (10^9^/L) )			0.315
<217.0	33 (24.1)	Reference	
217.0–252.5	21 (17.2)	0.71 (0.37–1.33)	
252.5–290.5	32 (20.6)	0.92 (0.51–1.64)	
≥290.5	24 (17.4)	0.69 (0.36–1.31)	

Abbreviations: SR, Saudi riyal (currency unit of Saudi Arabia); HbA1c, glycated hemoglobin; HDL, high-density lipoprotein cholesterol; LDL, low-density lipoprotein cholesterol; CRP, C-reactive protein.

† Logistic regression model adjusted for sex, age (continuous) and nationality (Saudi, non-Saudi).

‡ For ordinal variables, *P*-value was estimated from the linear trend test.

By using a logistic regression model with backward variable selection, the independent correlates were identified (Table 3). Every 5-year increase of diabetes history was associated with an OR of 1.91 for DPN (95% CI, 1.53–2.40). Abdominal obesity was associated with an OR of 2.53 for DPN (95% CI, 1.41–4.55), while oral hypoglycemic medication use was associated with an OR of 0.47 for DPN (95% CI, 0.26–0.85). The ORs for DPN associated with every 1-unit increment in fasting blood glucose, creatinine, and WBC were 1.05 (95% CI, 0.99–1.12), 1.07 (95% CI, 0.99–1.14) and 1.08 (95% CI, 1.01–1.16), respectively. In the sensitivity analysis, the backward variable selection algorithm identified the set of independent correlates except oral hypoglycemic medication use (Table S1).

**Table 3:** Backward multivariate logistic regression of risk factors associated with diabetic peripheral neuropathy. †

	Odds ratio	95% CI	*P* ‡
Sex, male vs. female	0.76	(0.44–1.29)	0.305
Age (years), every 1-year increase	1.04	(1.01–1.06)	0.008
Nationality, Saudi vs. non-Saudi	0.57	(0.35–0.94)	0.027
Abdominal obesity, case vs. non-case	2.53	(1.41–4.55)	0.002
Oral hypoglycemic, user vs. non-user	0.47	(0.26–0.85)	0.012
Duration of diabetes, every 5-year increase	1.91	(1.53–2.40)	<.001
Fasting blood glucose, every 1-mmol/L increase	1.05	(0.99–1.12)	0.082
Creatinine, every 10-ìmol/L increase	1.07	(0.99–1.14)	0.076
White blood cell, every 10^6^/L increase	1.08	(1.01–1.16)	0.021

† Logistic regression model adjusted for sex, age (continuous), nationality (Saudi Arabia, non-Saudi Arabia), abdominal obesity (case, non-case), Oral hypoglycemic (user, non-user), duration of DM (every 5 years), fasting blood glucose (every 1 mmol/L), creatinine (every 10 ìmol/L), and White blood cell (every 10^6^/L).

‡ For ordinal variables, *P*-value was estimated from the linear trend test.

## Discussion

In this large Saudi population, several independent correlates for DPN, in addition to blood glucose control and diabetes duration, were identified, including abdominal obesity, plasma creatinine and WBC levels.

The observed prevalence of DPN was 19.9% (95% CI, 16.7%–23.5%) in this diabetic population, which was higher than a worldwide estimate of DPN prevalence among diabetics (8.1% – 12.2%) [23]. In Saudi Arabia, a prevalence of 65.3% has been previously reported for painful DPN in a nationally representative diabetic population. [12] In other Middle East countries, the prevalence rates of painful DPN were 61.3%, 57.5%, 53.9% and 37.1% for Egyptian, Jordanian, Lebanese, and Gulf States population, respectively. [24] However, DPN cases in these two studies was ascertained by questionnaire (Douleur Neuropathique 4, DN4) other than objective measurement and the difference between our estimates and those from the previous studies in Middle East countries might be explained by discrepancy on ascertainment tools and definitions of DPN, which has been noticed by previous study [25]. A study based on a nationally representative US population with diabetes that also employed monofilament testing to detect DPN reported a prevalence of 28.5% [25]

Previous studies [3], [4], [5] and clinical guidelines [1], [26] have indicated aggressive blood glucose control as a standard clinical practice in the management of DPN. We observed that a lower level of fasting blood glucose and oral hypoglycemic use was associated with lower odds of DPN, which emphasizes the role of intensive glycemic control in DPN prevention and treatment. However, in our univariable analysis, we found that insulin use, another glycemic control medication, was associated with a 3.93-fold odds of DPN. A similar association between insulin use and DPN has been reported in previous studies [5], [6]. In this study context, insulin use tends to be an indicator for longer duration and greater severity of diabetes. Consistent with previous findings [4], [5], [27], [28], our study also observed that a strong association between longer duration of diabetes and DPN. Additionally, the significantly higher odds of DPN in type 1 diabetic participants observed in the univariable analysis could be ascribed to the longer course of disease in type 1 diabetics. Even though not a modifiable risk factor, duration of diabetes is of great importance for early identification and management of DPN. Previous studies have suggested that elevated level of HbA1c, a maker for long-term chronic glycemic exposure, strongly predicted risk of DPN [5], [6], [27], [28], [29], although our study did not observe a significant association between HbA1c and DPN, especially after adjusting for duration of diabetes.

Our data suggested that abdominal obesity was a significant and independent correlate for DPN, whereas general obesity (BMI ≥30 kg/m^2^) was not significant after multivariate adjustment. This finding is not surprising given that insulin resistance is more strongly related to abdominal obesity than general obesity [30]. Previous studies have suggested an association between insulin resistance with impaired autonomic function and occurrence of DPN [31]. Existing evidence regarding the association between abdominal obesity and DPN was sparse and inconsistent. Some previous studies addressed the association between abdominal obesity and peripheral neuropathy (PN) in study populations including both diabetics and non-diabetics, and reported an positive association between waist circumference and PN [32], [33]. In a community-based Australian population, no significant association between waist circumference and DPN was observed in either newly diagnosed or prevalent diabetic populations [34].

Considering aggressive treatment of hypertension and dyslipidemia has become standard clinical practices in the management of diabetic nephropathy and retinopathy, emerging evidence has suggested that hypertension and dyslipidemia could also be new targets for both DPN prevention and treatment [5], [35], [36]. Our study found a significant association between statin use and DPN, but non-significant association between lipid profile and DPN. These findings could be attributed to a high prevalence of statin use in this population (38.5%). Statin was likely to be a surrogate for dyslipidemia in this population. In this study, hypertensive participants were more prone to have DPN when comparing to normotensive participants in the univariable analysis, which was consistent with previous findings [5], [6], [37]. However, this association disappeared in the multivariable analysis.

This current analysis observed an elevated level of CRP, a circulating maker of inflammation, among DPN cases. Our finding is consistent with a previous report from Herder et al., in which a high level of CRP was found to be associated with diabetic polyneuropathy and some neuropathic deficits [38]. Similarly, increased CRP level has also been observed among diabetic foot ulcer patients [39]. However, after multivariable adjustment CRP was not an independent correlate for DPN in the study, while Herder et al. found an independent and persistent association between CRP and polyneuropathy even after multivariable adjustment. Another biomarker of inflammation, WBC count, was independently and positively associated with the presence of DPN in our analysis. To our knowledge, our analysis is the first epidemiological study that demonstrated this independent association. A recent breakthrough of pathological mechanism of DPN was the finding of inflammatory infiltrates around epineurial and perineurial blood vessels from biopsy specimen, suggesting inflammatory process was responsible for occurrence of DPN in addition to nerve ischemia [23]. Moreover, a previous study has demonstrated that accumulation of advanced glycation end products (AGEs), caused by long-term exposure to hyperglycemia, correlated with the severity of peripheral and autonomic nerve abnormalities in diabetes even before being clinically manifest; the AGE–RAGE (receptor for AGE) interaction was a propagating factor for chronic inflammation [40]. As clinical markers for renal function, creatinine and serum urea nitrogen were related to DPN in this study. In addition, creatinine was identified as an independent correlate for DPN. This finding is consistent with previous studies [41], [42]. Nephropathy, as another common diabetes complication, is often concomitant with DPN, accounting for the elevated levels of these two clinical markers of renal function [37].

We also observed a strong positive association between elevated level of homocysteine, an indicator of oxidative stress, and DPN in the univariable analysis, but this association disappeared in our multivariable analysis. A increasing body of evidence has suggested the important role of oxidative stress in the pathogenesis of diabetic neuropathy in animal models [23]. In addition, a clinical trial in a German population found that α-lipoic acid, a powerful antioxidant, had positive effect on alleviating neuropathic symptoms, also providing supporting evidence for the relationship between oxidative stress and DPN [43]. It is of interest that a negative association between hemoglobin level and DPN was found in this study. We regarded hemoglobin level as an indicator for severity of diabetes in this context, since previous study has already found anemia was a common accompaniment to diabetes, as well as a negative association between severity of diabetes and hemoglobin level [44]. However, the possibility that hemoglobin may be an indicator for hypoxic damage cannot be ruled out, since that microvascular damage caused by long-term exposure to hyperglycemia may lead to diminished blood flow to nerve tissues vulnerable to hypoxic damage, and thereby to the development of neuropathy [45].

To our knowledge, this is the first study on correlates of DPN in a Saudi population. The strengths of this study include a large sample size and a detailed and objective assessment of DPN. Combining the validated tests for superficial and deep sensations and neurological symptoms, the diagnosis procedure had enough sensitivity in detecting DPN [46], [47]. In addition, comprehensive measures of clinical and biochemical markers were assessed. However, several limitations of this study warrant attentions. First, the cross-sectional nature of this study design limits the inference of causal relationship between correlates and DPN. Therefore, our findings need to be confirmed in prospective studies. Secondly, because we used advertisement to recruit participants, eligible participants with severe potential risk factors might not be able or willing to participate in this study. Thus, findings from this study may not be generalizable to other populations. Thirdly, since the diagnosis of DPN was only based on a combination of decreased sensation and neuropathic sensory symptoms, without nerve conduction test[26], possible misclassification of DPN diagnosis cannot be ruled out. However, previous study has found a relatively high sensitivity of the criteria employed by the current study (>87%) [1]. Therefore, the misclassification is likely to be small. Lastly, even though we imputed missing values for clinical markers by their median values, potential selection bias introduced by imbalance between participants with and without missing values could not be ruled out. However, the characteristics of participants with and without missing values were generally balanced (data not shown), indicating that the likelihood of selection bias was relatively small.

In conclusion, a higher prevalence of DPN was observed in this Saudi population with diabetes, compared to the worldwide average estimate. In line with previous findings, diabetes duration and glycemic control were strongly associated with DPN. Other correlates, including abdominal obesity and two relatively novel clinical markers (creatinine and white blood cell count) were also identified, which may contribute to the risk prediction of DPN. Furthermore, targeting patients with high risk of DPN in foot education programs may have important clinical implication in preventing foot ulceration and subsequent lower-extremity amputation, especially in countries, like Saudi Arabia, with high disease burden of diabetes. To further address prediction of DPN risks in Saudi populations with diabetes, prospective cohort studies are still warranted.

## Supporting Information

Table S1 **Backward multivariate logistic regression of risk factors associated with diabetic peripheral neuropathy among participants with type 2 diabetes.**
(DOCX)
