# Supplementary material for: Prevalence and Correlates of Diabetic Peripheral Neuropathy in a Saudi Arabic Population: A Cross-Sectional Study
Source: PLoS One. 2014 Sep 3;9(9):e106935. doi: 10.1371/journal.pone.0106935 (PMC4153691; doi:10.1371/journal.pone.0106935)
Supplement: Table S1 — Backward multivariate logistic regression of risk factors associated with diabetic peripheral neuropathy among participants with type 2 diabetes. (DOCX) [file pone.0106935.s001.docx]

Table S1 Backward multivariate logistic regression of risk factors associated with diabetic peripheral neuropathy among participants with type 2 diabetes ^†^

|  | Odds ratio | 95% CI | *P* ^‡^ |
| --- | --- | --- | --- |
| Sex, male vs. female | 0.78 | (0.45-1.35) | 0.372 |
| Age (years), every 1-year increase | 1.03 | (1.00-1.06) | 0.051 |
| Nationality, Saudi vs. non-Saudi | 0.62 | (0.37-1.02) | 0.059 |
| Abdominal obesity, case vs. non-case | 2.47 | (1.34-4.57) | 0.004 |
| Duration of diabetes, every 5-year increase | 2.01 | (1.59-2.54) | <.001 |
| Fasting blood glucose, every 1-mmol/L increase | 1.05 | (0.98-1.11) | 0.154 |
| Creatinine, every 10-μmol/L increase | 1.10 | (1.02-1.18) | 0.011 |
| White blood cell, every 10^6^/L increase | 1.07 | (1.00-1.15) | 0.050 |

^†^ Logistic regression model adjusted for sex, age (continuous), nationality (Saudi Arabia, non-Saudi Arabia), abdominal obesity (case, non-case), Oral hypoglycemic (user, non-user), duration of DM (every 5 years), fasting blood glucose (every 1 mmol/L), creatinine (every 10 μmol/L), and White blood cell (every 10^6^/L).

^‡^ For ordinal variables, *P*-value was estimated from the linear trend test.
